# Yoga Effects on Anthropometric Indices and Polycystic Ovary Syndrome Symptoms in Women Undergoing Infertility Treatment: A Randomized Controlled Clinical Trial

**DOI:** 10.1155/2021/5564824

**Published:** 2021-06-10

**Authors:** Maryam Mohseni, Mohammad Eghbali, Homa Bahrami, Farzaneh Dastaran, Leila Amini

**Affiliations:** ^1^School of Nursing and Midwifery, Iran University of Medical Sciences, Tehran, Iran; ^2^Department of Nursing, University of Social Welfare and Rehabilitation Sciences, Tehran, Iran; ^3^Torbat Heydariyeh University of Medical Sciences, Torbat Heydariyeh, Iran; ^4^Sarem Fertility and Infertility Research Center (SAFIR), Sarem Women's Hospital, Tehran, Iran; ^5^School of Nursing and Midwifery, Tehran University of Medical Sciences, Tehran, Iran; ^6^Nursing Care Research Center (NCRC), Iran University of Medical Sciences, Tehran, Iran; ^7^Department of Midwifery, School of Nursing and Midwifery, Iran University of Medical Sciences, Tehran, Iran

## Abstract

The aim of this study was to investigate the effects of yoga exercises on anthropometric parameter and clinical sign of PCOS among women undergoing infertility treatment. This clinical trial study was performed on 61 women with PCOS who have undergone infertility treatment at Sarem Hospital in Tehran, Iran. The patients were first selecting based on purposeful and then randomly assigning to the intervention and control groups. In the intervention group, yoga exercises were performed for 6 weeks and the patients in the control group only received routine care. Anthropometric parameters and clinical signs were performed and recorded. After the intervention, here was a significant reduction in hirsutism, abdominal circumference, and hip circumference scores in the intervention group compared to the control group (*P* < 0.05). Given the effects of yoga exercises on the improvement of hirsutism, abdominal circumference, and hip circumference, it is suggested to use yoga as a treatment strategy in women with PCOS.

## 1. Introduction

Polycystic ovary syndrome (PCOS) is one of the most common endocrine disorders among women in their reproductive age and the prevalence of this syndrome varies from 6.5 to 19.9% in the world [1, 2]. According to the latest systematic review studies, the prevalence of this syndrome is estimated at 3% according to the definition provided by the National Institutes of Health (NIH) and at 7% according to the definition provided by Rotterdam in Iran [3].

Polycystic ovary syndrome is the most leading cause of ovulation disorders and one of the most important causes of infertility [4]. Infertility is considered as one of the most common problems in human societies which has affected between 60 and 80 million couples worldwide according to the World Health Organization reports [5]. The prevalence of infertility in Iran is reported to be about 7.88% [2, 5].

Polycystic ovary syndrome can increase androgen level (sings such as acne, hirsutism, and alopecia) and nonovulatory characteristics (such as oligomenorrhea, amenorrhea, or infertility) [2]. Diagnostic criteria for this syndrome include lack of ovulation, clinical or laboratory hyperandrogenism, or observation of polycystic ovaries on ultrasound [6].

Different mechanisms involved in the pathogenesis of polycystic ovary syndrome have led to various treatment approaches for this disease [7]. Although pharmaceutical treatments have been suggested for this disease, there are no signs of safety and security for these drugs, especially for the long-term use. In addition, none of these drugs have been approved by the American Food and Drug Administration to use for polycystic ovary syndrome [8].

It can be said that lifestyle modification is one of the most important and safest treatments that has always been considered as the first step in treatment. Lifestyle modification includes a combination of behavioral (reducing psychosocial stressors), nutritional, and exercise therapies [8, 9]. Lifestyle modifications include dietary changes and exercise activities, which help reduce the prevalence of obesity and hormonal problems among these individuals [10]. Physical activity along with weight loss can reduce the level of insulin as well as androgens and can improve the patients' symptoms [11]. It has been well established that weight loss by 5–14% through energy restriction and physical activity can lead to the improvement of reproductive function and hormonal characteristics among obese and overweight women with polycystic ovary syndrome [12].

Yoga is one of the recommended exercises which can lead to the improvement of this disease. It is an important factor in improving physical and mental symptoms, especially emotional health. It is noteworthy that doing yoga exercise does not require physical fitness or flexibility [13]. Physical and respiratory yoga exercises help increase flexibility and muscular strength and can also improve blood circulation and oxygen delivery to all the cells and reproductive tissues [14]. Moreover, relaxation and comforting thoughts in yoga improve the autonomic nervous system and reduce blood pressure and triglycerides, regulate breathing, and control emotions [15]. Yoga improves reproductive function by regulating the endocrine system, reducing stress, and balancing neuronal hormones. Yoga also helps reduce serum cortisol by increasing cortisol excretion, which in turn can improve the symptoms of PCOS [13].

Today, yoga is used as a treatment for various conditions such as hypertension, multiple sclerosis, asthma, low back pain, and arthritis, as well as pain and stress management. The results of various studies have shown that doing yoga exercises in women with polycystic ovary syndrome has led to improved insulin sensitivity and lipid indexes [16–19]. Furthermore, in the latest systematic review studies conducted in 2020, it was stated that although regular exercises have helped improve hormonal, metabolic, and clinical parameters among these patients, there is the need for better and more accurate studies in this regard [8].

Since midwives are responsible for the prevention of disease complications and the promotion of reproductive health, especially through complementary medicine approaches and lifestyle changes, it seems that examining the effect of exercise in women with reproductive problems such as infertile women with polycystic ovary syndrome is considered as one of the priorities of reproductive health. In addition, given the limited existing studies on the effects of yoga exercises on polycystic ovary syndrome, the present study aims to determine the effect of yoga exercises on anthropometric parameters and clinical signs among women with polycystic ovary syndrome undergoing infertility treatment.

## 2. Methods

### 2.1. Design

This randomized control clinical trial study was conducted with an intervention and a control group in parallel. The study was registered at the Iranian clinical trial system (registration no. IRCT2016012326158N1).

### 2.2. Population and Sampling

Women with PCOS who referred to Sarem Hospital in Tehran, Iran, with the aim of starting the IVF infertility treatment cycle between March 2016 and November 2016 were asked to participate in this study. The inclusion criteria for the present study were (1) diagnosis with PCOS and undergoing infertility treatment, (2) no history of yoga exercises, (3) no history of debilitating chronic illness, (4) no drug addiction and smoking history, and (5) no experience of stressful events in the past three months. Moreover, the patients would be excluded if they skip the yoga trainings for more than one day a week and also in case of the occurrence of any acute physical or mental stressors during the study. Polycystic ovary syndrome was assumed to be diagnosed by a gynecologist according to the Rotterdam scale which was proposed in 2003. It is noteworthy that women with at least two of the amenorrhea or oligomenorrhea criteria, laboratory or clinical hyperandrogenism, or observation of polycystic ovaries on ultrasound were identified by the physician as the patients with PCOS [20].

Eligible individuals were selected purposefully and constantly and were then randomly assigned into intervention and control groups. A randomized block design was used for randomization. Accordingly, four letters of A, B, C, and D were written in 4 similar envelopes where A and C belonged to the intervention group and B and D belonged to the control group; besides, the arrangement of cards was performed according to different permutations and four people were randomly selected based on the multiples of 4, and the first to fourth persons would pick up a card, respectively. They were eventually assigned to one of the intervention or control groups according to the type of the cards.

According to the researches done in similar fields and given the 90% confidence level and 80% power, the sample size was calculated with 30 people in each group (60 people in total); in addition, considering the 10% probability of sample drop, finally 67 people were included in this study.

### 2.3. Instruments and Data Collection

The data were collected through a demographic information questionnaire and also information about the menstrual cycle using a checklist for recording clinical signs and anthropometric parameters.

#### 2.3.1. Demographic Information

Demographic information and information about the menstrual cycle were completed through interviews with patients. The demographic information questionnaire includes age, level of education, occupation, economic status, and insurance status. Menstrual cycle information included the age at the first menstruation, duration of each cycle, menstrual pain, and duration of infertility.

#### 2.3.2. Anthropometric Parameter and Clinical Sign

The data on clinical signs and anthropometric parameters were collected through a researcher-made checklist. The checklist included information on body mass index, abdominal and hip circumference, systolic and diastolic blood pressure, acanthosis nigricans, alopecia, and hirsutism. Content validity based on the opinion of 10 faculty members was measured to determine the scientific validity of this checklist. Moreover, intraclass correlation coefficient (ICC) test was used to evaluate the reliability of the checklist, which was calculated at 0.87. Measurement of the anthropometric parameters and also the clinical signs of PCOS were performed as follows.

The patients' weight was measured at a specific time of the day (morning) with minimal body cover (disposable scrub) using an accurate digital scale. In order to measure height, the person should stand upright next to and completely attached to a wall; besides, the patient was in a position where the head was straight forward, and then the distance between the top of the head and the floor was measured. Body mass index was also determined by dividing their weight (kg) by their height (meters) to the power of 2.

Abdominal circumference was calculated using a standard meter at an equal distance from the lower edge of the ribs and below the upper edge of the pelvis around the umbilicus in the middle of the iliac crest, in a position where the breath was not trapped and the abdomen was not hold inside. To measure the hip circumference, the widest part of the hip was calculated considering that the hip was not contracted. To avoid possible errors, all the measurements were performed before and after the intervention by the same person, i.e., the researcher; and the researchers attempted to ensure that the measuring tape was neither too loose nor too tight around the abdomen and hips. The researcher also made sure that the tape was not twisted around the abdomen and the waist.

Systolic blood pressure and diastolic blood pressure were measured through the monitoring procedure for all patients in the hospital. All measurements were calibrated in terms of measurement accuracy by the medical engineer in the hospital and all the measurements were performed by the researcher.

Three signs of *Acanthosis nigricans*, alopecia, and hirsutism were recorded through observation. Hirsutism was assessed using the Ferriman–Gallwey (mFG) scoring system, a system of scoring androgen-sensitive hair in nine areas of the body including midline hair, beard line, mustache, beard, breast hair, and the hair inside the thigh area and the middle line of the lower back and the arm. The scale was rated between zero (no hair) and 4 (completely hairy), where the overall score above 7 is defined as hairy [21, 22]. The score was assessed by the researcher through observation of these nine areas and based on the images of the mFG checklist.

### 2.4. Study Procedures

**Figure 1:**
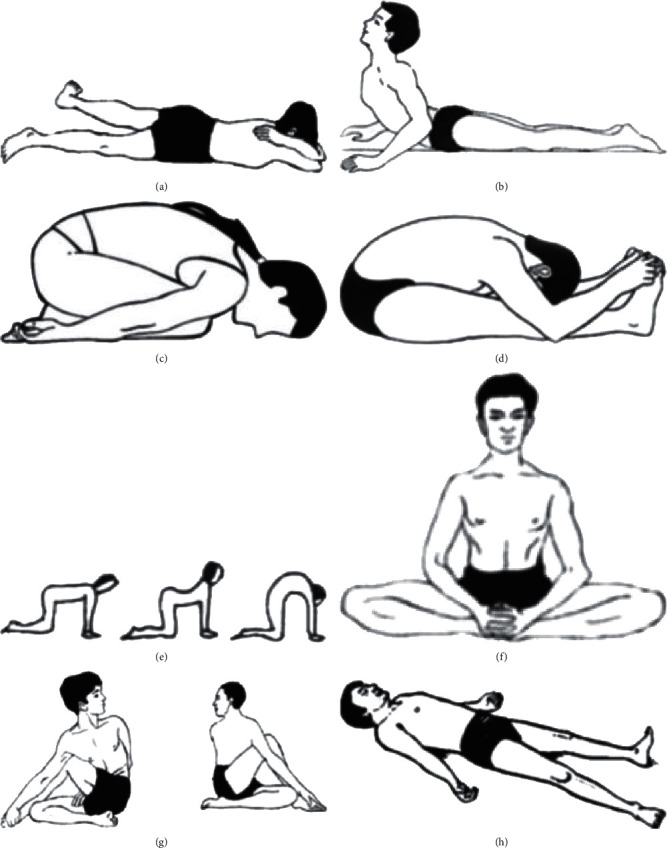
Images of the different asana positions in yoga session for 45 minutes: (a) makarasana; (b) bhujangasana; (c) dharmikasana; (d) paschimottanasana; (e) sardulasana; (f) bhadrasana; (g) matsyedrasana; (h) savasana according to [23–25].

After obtaining the code of ethics and the necessary permissions to start sampling, the researcher referred to the research environment with a letter of introduction. After identification of the eligible patients, they were provided with sufficient information about the objectives of the research and the study procedure, and informed written consent was obtained from the volunteer participants. The researcher also informed the participants that they may randomly be assigned to the intervention or control group. In addition to receiving routine care, the participants in the intervention group were asked to perform yoga exercises for 6 weeks. Yoga exercises were taught by the instructor for 90 minutes and also each participant was given a training package which was pre-prepared by the researcher. These training sessions were then performed twice a week by the instructor at the gym inside the hospital as well as another 5 sessions per week at home by the participants themselves. The training sessions were performed as follows: at first, the participants would do 25 minutes of exercises in order to relieve physical stress, followed by 45 minutes of asana exercises, and the last 20 minutes were dedicated to deep relaxation exercises (Figure 1). The researcher would call the participants five times a week so as to follow up on application of the yoga exercises according to the research protocol. The participants in the control group only received routine care which was provided by the hospital during this period, and the timing of the two groups was planned in such a way that there was no interference between them.

Moreover, anthropometric parameters, blood pressure, and clinical signs were measured once before the intervention and then 6 weeks after the intervention and the data were recorded in the checklist accordingly.

### 2.5. Data Analysis

The data were analyzed using SPSS software version 22. In addition, any level of lower than 0.05 was considered significant for all the analyses. Statistical tests such as ANCOVA, independent *t*-test, chi-square, and Fisher's exact test were also conducted.

### 2.6. Ethical Considerations

This study is a part of a master's thesis that has been approved by Iran University of Medical Sciences and Health Services. It was then evaluated and approved by the ethics committee (no. IR.IUMS.REC.1394.9311373025). All the participants were informed of the objectives of the study and also informed written consent was obtained. The participants were then assured that they could leave the study at any stage of the study and that this intervention would not interfere with their normal healing process. In addition, after completing the study, the training packages were also delivered to the participants in the control group as they did not receive yoga exercises during the study.

## 3. Results

### 3.1. Follow-Up

**Figure 2:**
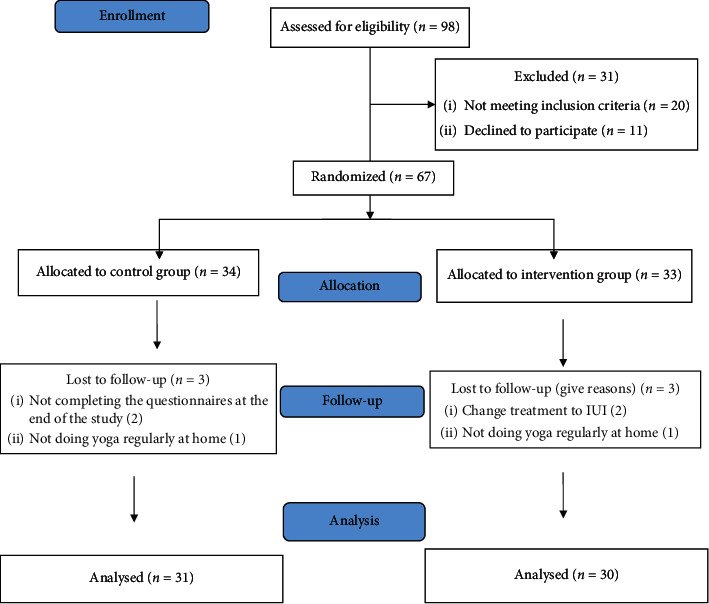
Study design flow diagram.

Only 67 of all the 98 patients that were initially evaluated were eligible for the resent study and were then randomly assigned to the intervention and control groups. In the intervention group, 2 patients were excluded from the study due to the changes in the treatment and also another patient due to irregular performance of yoga exercises. Besides, two patients were excluded from the study due to not completing the questionnaires at the end of the study and also another patient was excluded due to irregular performance of yoga exercises in the control group (see Figure 2).

### 3.2. Demographic Information and Menstrual Cycle Characteristics

The mean and standard deviation of age was 30.77 ± 6.01 years and 30.35 ± 5.53 in the intervention and the control group, respectively; however, there was no significant difference observed between the two groups (*P*=0.782). Moreover, university degree was reported as the highest level of education in both intervention and control groups (56.7% in the intervention group and 61.3% in the control group). The age of the first menstrual period was 13 ± 1.53 in the intervention group and 12.9 ± 1.72 in the control group. Nonetheless, there was no statistically significant difference between the two groups in terms of demographic factors and menstrual cycle (see Table 1).

### 3.3. Anthropometric Parameter

Anthropometric parameters include body mass index and abdominal and hip circumference, as well as systolic and diastolic blood pressure in the two groups once before the intervention and then 6 weeks after the intervention. Based on the findings, there was no statistically significant difference between the two groups in terms of body mass index and abdominal circumference, as well as systolic and diastolic blood pressure before the study, so the two groups were homogeneous. However, there was a significant difference between the two groups in terms of hip circumference (*P*=0.029). Since the mean of the hip circumference was different in the two groups before the study, analysis of covariance was used for comparison after the intervention. The results of the management of the average hip circumference before the intervention showed that there was a statistically significant difference in hip circumference (*P*=0.027) between the two groups after the intervention; besides, the reduction of the hip circumference was higher in the intervention group. Moreover, there was a significant difference between the two groups in terms of abdominal circumference six weeks after the intervention (*P*=0.045) and the mean was lower in the intervention group. On the other hand, there was no statistically significant difference between the two groups in terms of body mass index (*P*=0.929), systolic pressure (*P*=0.402), and diastole (*P*=0.245) after the intervention (Table 2).

### 3.4. Clinical Signs

According to the data in Table 3, the two groups were not statistically significant in terms of *Acanthosis nigricans*, alopecia, and hirsutism at the beginning of the study; so, there was homogeneity between the two groups. Nonetheless, based on the results of the independent *t*-test after 6 weeks of yoga exercises, there was a statistically significant difference between the two groups in terms of the Ferriman–Gallwey test scores (hirsutism) (*P* < 0.001). In contrast, there were no statistically significant differences between the two groups regarding the other symptoms of *Acanthosis nigricans* and alopecia (*P*=0.999) and (*P*=0.949), respectively.

## 4. Discussion

The present study was conducted with the aim of evaluating the effectiveness of 6 weeks of yoga exercises on anthropometric parameters and clinical signs among women with polycystic ovary syndrome who were undergoing infertility treatment in Iran. According to the findings, practicing yoga for 6 weeks helped participants in the intervention group reduce the abdominal circumference, hip circumference, and hirsutism score compared to those in the control group who had only received the routine care.

Previous studies have examined various yoga exercises on patients with polycystic ovary syndrome. But, to the best of the researchers' knowledge, no study has examined the effect of 6 weeks of yoga practice on clinical signs and anthropometric parameters so far. In this regard, the results of the present study showed that yoga exercises have only led to a significant improvement in hirsutism among the patients in the intervention group. However, there was no significant change in other clinical signs such as alopecia and *Acanthosis nigricans*. Accordingly, the results of another study which was conducted by Nidhi et al. to evaluate the effect of comprehensive yoga exercises on endocrine parameters among the adolescents showed that 12 weeks of yoga exercises have significantly reduced the rate of hirsutism in these patients [26]. Patel et al. also examined the effectiveness of yoga mindfulness exercises for three months; they found that the intervention had no effects on hirsutism. This could be contributed to the type and intensity of the exercises [13].

The findings of the present study regarding the anthropometric parameters of polycystic ovary syndrome showed that performing yoga exercises for 6 weeks can lead to a significant decrease in abdominal and hip circumference. However, Yoga trainings did not have any significant effects on systolic and diastolic blood pressure and body mass index. In a seminal study by Wolff et al., the effect of yoga exercises was investigated on blood pressure and quality of life in patients with hypertension in 2013; the results showed that performing yoga exercises in the classroom did not lead to a significant reduction in systolic and diastolic blood pressure in those patients (*P* > 0.05) [27]. On the other hand, the results of another study by Lakkireddy et al. on the effect of yoga exercises in patients with cardiac fibrillation indicated a significant difference in systolic and diastolic blood pressure after 3 months of yoga exercise [28]. Placing the body in different positions during yoga trainings may affect the functions of the sympathetic system by activating the baroreflex and reduce blood pressure, heart rate, and catecholamine [29]. In addition, since yoga is considered as an aerobic exercise, the effects of aerobic exercises on lowering systolic and diastolic blood pressure can also be observed in yoga trainings. It seems that the reason for the inconsistency with the results of the present study is the nature of the disease of the patients under study as well as the severity and duration of yoga.

Vanitha et al. attempted to investigate the effect of yoga exercises on cardiovascular parameters in women with PCOS; and the results showed that performing yoga exercises for 12 weeks can lead to a significant decrease in weight and body mass index. The different findings can be contributed to the duration of the exercises as well as the difference in the type of yoga exercises [30].

### 4.1. Clinical Implication

The findings of the present study led to the improvement of our knowledge regarding the beneficial effects of asana yoga training on clinical signs and anthropometric parameters among infertile women with PCOS. Given the importance of polycystic ovary syndrome and the severity of its common complications, especially infertility and different impacts on various aspects of people's lives, it is necessary for the specialists such as gynecologists and midwives to provide solutions. Therefore, it is recommended that the instruction of complementary medicine methods such as yoga be considered by educational planners and politicians in order to reduce the complications of polycystic ovary syndrome.

### 4.2. Strengths and Limitations

To the best of our knowledge, this study was one of the pioneering studies to investigate the effects of yoga (asana) exercises on PCOS signs in women undergoing infertility treatment. However, the interpretation of such results and its generalization should be done with caution because this study has been done in a small community of women in Iran. On the other hand, blindness was not applied in this study. It is also noteworthy that, despite employing a standard measurement scale as well as an educated expert (midwife) to conduct the measurements and to record the data, there might be some bias within the process. Thus, it is suggested to consider the findings with more caution. Due to the limited availability of the samples and the infertility treatment cycle, the study was conducted in a short duration of time which was considered as one of the serious limitations of this study. Therefore, it is suggested that future studies be conducted for a longer period of time. Meanwhile, having a control group and using block random sampling were the strengths of this study. This study can also be considered as a framework for other studies in the field of obstetrics and polycystic ovary syndrome, infertility, and complementary medicine. The effects of yoga exercise on anthropometric parameters and clinical signs of PCOS have also been reported inconsistently in various studies; hence, it is suggested to investigate the effects of yoga exercises on a larger sample of infertile women or on treatment outcomes in women with other causes of infertility.

## 5. Conclusion

The findings of the present study showed that yoga (asana) exercises for 6 weeks and 90 minutes per session were effective in improving some clinical signs (hirsutism) as well as anthropometric parameters (abdomen and hip circumference) in infertile women. Based on the results of this study, it is suggested that health care personnel, midwives, and gynecologists recommend this method to women with PCO along with other treatments. However, it is also suggested that future studies be performed to evaluate such treatment with a larger sample size and longer duration of intervention (e.g., 12 weeks of yoga trainings). Moreover, the use of different methods of yoga exercises is also recommended in order to control the symptoms of PCOS.

## Figures and Tables

**Table 1:** Sociodemographic and menstrual cycle characteristic of all patients.

Variable	Intervention group (*N* = 30) mean ± SD	Control group (*N* = 31) mean ± SD	*P* value
Age	30.77 ± 6.01	30.35 ± 5.53	0.782^*∗*^
Level of education			
Middle school	4 (13.3%)	7 (22.6%)	0.358^*∗∗*^
Diploma	9 (30%)	5 (16.1%)	
University	17 (56.7%)	19 (61.3%)	
Occupation			
Housewife	19 (63.3%)	16 (51.6%)	0.355^*∗∗*^
Employee	11 (36.7%)	15 (48.4%)	
Economic status			
Desirable	9 (30%)	10 (32.3%)	0.999^*∗∗∗*^
Partly desirable	20 (66.7%)	20 (64.5%)	
Undesirable	1 (3.3%)	1 (3.2%)	
Insurance status			
Yes	28 (93.3%)	28 (90.3%)	0.999^*∗∗∗*^
No	2 (6.7%)	3 (9.7%)	
Menstrual pain			
Mild	10 (33.3%)	15 (48.4%)	0.169^*∗∗∗*^
Moderate	17 (56.7%)	16 (51.6%)	
Severe	3 (10%)	0	
Age at the first menstruation (years)	13 ± 1.53	12.9 ± 1.72	0.817^*∗*^
Duration of each cycle (days)	31.77 ± 5.38	31.13 ± 5.69	0.655^*∗*^
Duration of infertility (years)	6.60 ± 2.71	6.97 ± 3.75	0.664^*∗*^

^*∗*^Independent *t*-test. ^*∗∗*^Chi-square test. ^*∗∗∗*^Fisher exact test.

**Table 2:** Anthropometric parameter of PCO in the intervention group and control group.

	Intervention group, *N* = 30, mean (SD)	Control group, *N* = 31, mean (SD)	*P* value
Body mass index			
Baseline	25.96 (3.24)	25.50 (3.50)	0.597^*∗*^
6 weeks later	25.32 (2.63)	25.39 (3.50)	0.929^*∗*^
Abdominal circumference (cm)			
Baseline	93.83 (8.29)	94.97 (7.61)	0.580^*∗*^
6 weeks later	87.23 (8.29)	91.55 (8.18)	0.045^*∗*^
Hip circumference (cm)			
Baseline	102.97 (10.24)	98.06 (6.26)	**0.029** ^*∗∗*^
6 weeks later	95.80 (9.14)	94.84 (8.38)	0.027^*∗∗*^
Systolic blood pressure (mmhg)			
Baseline	110.23 (10.33)	110.26 (10.15)	0.938^*∗*^
6 weeks later	110.00 (10.26)	110.29 (1.42)	0.402^*∗*^
Diastolic blood pressure (mmhg)			
Baseline	60.73 (10.34)	70.26 (10.06)	0.096^*∗*^
6 weeks later	70.20 (0.89)	70.48 (0.99)	0.245^*∗*^

^*∗*^Independent *t*-test. ^*∗∗*^ANCOVA.

**Table 3:** Clinical sign of PCO in the intervention group and control group.

	Intervention group, *N* = 30, mean (SD)		Control group, *N* = 31, mean (SD)		*P* value
	Yes	No	Yes	No	
*Acanthosis nigricans*					
Baseline	4 (13.3%)	26 (86.7%)	4 (12.9%)	27 (87.1%)	0.999^*∗*^
6 weeks later	4 (13.3%)	26 (86.7%)	4 (12.9%)	27 (87.1%)	0.999^*∗*^
Alopecia					
Baseline	6 (20%)	24 (80%)	6 (19.4%)	25 (87.1)	0.949^*∗∗*^
6 weeks later	6 (20%)	24 (80%)	6 (19.4%)	25 (87.1)	0.949^*∗∗*^
Hirsutism (Ferriman–Gallwey)					
Baseline	8.87 (3.19)		10.19 (3.68)		0.139^*∗∗∗*^
6 weeks later	7.13 (2.14)		9.9 (2.02)		0.001^*∗∗∗*^

^*∗*^Fisher exact test. ^*∗∗*^Chi-square test. ^*∗∗∗*^Independent *t*-test.

## Data Availability

The data used to support the findings of this study are available from the corresponding author upon request.

## Supplementary Materials

Supplementary Materials Checklist for recording clinical signs and anthropometric parameters.
