# Supplementary material for: Yoga Effects on Anthropometric Indices and Polycystic Ovary Syndrome Symptoms in Women Undergoing Infertility Treatment: A Randomized Controlled Clinical Trial
Source: Evid Based Complement Alternat Med. 2021 Jun 10;2021:5564824. doi: 10.1155/2021/5564824 (PMC8213483; doi:10.1155/2021/5564824)
Supplement: Supplementary Materials — Checklist for recording clinical signs and anthropometric parameters. [file 5564824.f1.docx]

**Checklist clinical signs and anthropometric parameters**

Weight:

Height:

Body Mass Index:

Abdominal Circumference:

Hip Circumference:

Acanthosis nigricans: Yes 🞎 No🞎

Alopecia: Yes 🞎 No🞎

Ferriman-Gallwey (mFG) scoring:……………….

Systolic Blood Pressure:………………………….mmhg

Diastolic Blood Pressure:………………………….mmhg
